# An Extremely Rapid Case of Pneumonitis with the Use of Nivolumab for Pancreatic Adenocarcinoma

**DOI:** 10.1155/2018/6314392

**Published:** 2018-04-01

**Authors:** Rubens Barros Costa, Al Benson, Vahid Yaghmai, Ricardo L. B. Costa, Haijun Zhou, Amir Behdad, Jason B. Kaplan, Maureen Sadim, Sarah Talamantes, Aparna Kalyan

**Affiliations:** ^1^Developmental Therapeutics Program, Feinberg School of Medicine and Robert H. Lurie Comprehensive Cancer Center of Northwestern University, 233 East Superior Street, Chicago, IL 60611, USA; ^2^Division of Hematology and Oncology, Robert H. Lurie Comprehensive Cancer Center, Northwestern University, 676 North Clair Street, Chicago, IL 60611, USA; ^3^Department of Radiology, Northwestern University Feinberg School of Medicine, 676 North Saint Clair Street, Chicago, IL 60611, USA; ^4^Department of Breast Oncology, Lee Moffitt Cancer Center, Moffitt McKinley Outpatient Center, 10920 North McKinley Drive, BR-Program, Tampa, FL 33612, USA; ^5^Department of Pathology, Northwestern University Feinberg School of Medicine, 251 East Huron Street, Chicago, IL 60611, USA; ^6^Northwestern University Feinberg School of Medicine, 420 East Superior Street, Chicago, IL 60611, USA

## Abstract

Pancreatic cancer is the fourth most common cancer death in the United States despite comprising a small percentage of the total number of cancer cases. The estimated 5-year overall survival (OS) for patients with distant metastatic disease is approximately 3%. New treatment options are an unmet need and remain an area of active investigation. A 53-year-old male with metastatic pancreatic cancer presented to the hospital with acute-on-chronic respiratory failure approximately 24 hours after receiving a novel therapeutic combination. Chest imaging showed marked changes as concerning for pneumonitis. Infectious workup was negative. The patient had initial clinical improvement after receiving initial intravenous steroids and oxygen support but eventually deteriorated later opting for supportive measures only. With infection ruled out, drug-induced pneumonitis was felt to be the likely cause of the radiologic and clinical changes. The rapidity of onset of symptoms is the aspect being highlighted in this case.

## 1. Introduction

Pancreatic cancer is the fourth leading cause of cancer death in the United States and is expected to become the second leading cause of cancer death by 2020. It is estimated that approximately 53,670 patients will be diagnosed and 43,090 will die of pancreatic cancer in 2017. The estimated 5-year overall survival (OS) for patients with distant metastatic disease is approximately 3% [1].

Gemcitabine has historically been the backbone in the treatment of metastatic pancreatic cancer. In a randomized phase 3 clinical trial, gemcitabine was compared to 5-fluorouracil (5-FU) for the treatment of previously untreated metastatic pancreatic adenocarcinoma. The median OS and 12-month survival rate were 5.6 months and 18% for patients who received gemcitabine. These results led to FDA approval of gemcitabine for the treatment of metastatic pancreatic cancer in 1996 [2].

In a more recent phase II/III randomized trial, gemcitabine was compared to the combination regimen FOLFIRINOX (fluorouracil, irinotecan, and oxaliplatin). The median OS was 11.1 months for the combination compared with 6.8 months with gemcitabine monotherapy. Median progression-free survival (PFS) and objective response rate (RR) favored FOLFIRINOX compared with gemcitabine alone: 6.4 months and 31.6% versus 3.3 months and 9.4%, respectively (*p* < 0.001) [3].

The MPACT gemcitabine was also compared to the combination of gemcitabine and nab-paclitaxel in a large randomized phase III trial. The combination regimen improved RR (23 versus 7%), median PFS (5.5 versus 3.7 months), and OS (8.5 versus 6.7 months) but at the expense of increased toxicity [4].

These two combinations are widely used across the US for the treatment of metastatic/advanced pancreatic adenocarcinoma [5]. Despite representing incremental progress in this field, prognosis remains poor. Therefore, new therapeutic agents and combinations are urgently needed.

Herein, we present a case of fulminant and fatal pneumonitis in a patient who received a novel combination of nivolumab, gemcitabine, and nab-paclitaxel for the treatment of metastatic pancreatic carcinoma.

## 2. Case Report

The patient was a 53-year-old male with no past medical history who presented to the Emergency Department for evaluation of abdominal pain of one-month duration. His physical examination and vital signs were unremarkable. All his laboratory data were within normal limits.

A contrast-enhanced computed tomography (CT) of the abdomen and pelvis showed multiple pulmonary nodules, a small left pleural effusion, a cystic lesion in tail of the pancreas measuring 4.2 × 2.7 × 3.2 cm, and cystic hepatic lesions. Nonenhanced CT of the chest demonstrated a 4 cm pleural-based lung mass along the anterior medial aspect of the left upper lobe and innumerable indistinct nodular densities in both lungs and hepatic metastases.

**Figure 1:**
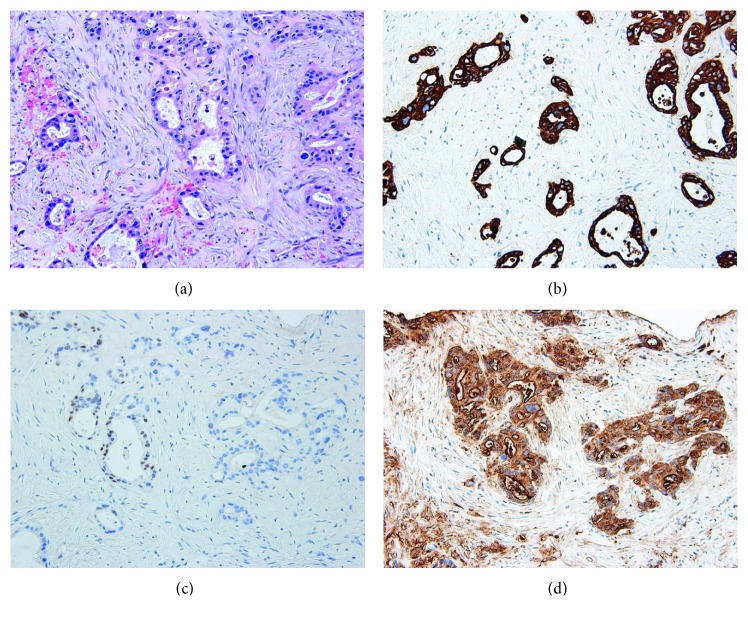
(a) Lung lesion histology (H&E, 200x); (b) cytokeratin-7 immunohistochemical stain; (c) immunohistochemical stain; (d) CA19-9 immunohistochemical stain (200x for all IHC stains).

A left upper lobe lung fine-needle aspirate and core biopsy revealed a malignant infiltrative proliferation of epithelial cells forming glands, indicating adenocarcinoma. The cells displayed nuclear enlargement, hyperchromasia, marked anisonucleosis, scant eosinophilic cytoplasm, and mitotic activity. The background stroma was fibrotic displaying desmoplasia and areas of necrosis. Immunohistochemical stains were as follows: CK7 positive, CA19-9 positive, CDX2 weakly focal positive, CK20 negative, and TTF1 negative. The cytomorphologic results were consistent with a metastatic process of pancreatic origin (Figure 1). The serum CA19-9 was elevated at 737 U/mL.

He was evaluated by the medical oncology service who recommended treatment with the FOLFIRINOX regimen. Three weeks later, he was seen at our institution for a second opinion and consideration of a clinical trial. After further discussion, he opted to participate in a clinical trial utilizing gemcitabine, nab-paclitaxel, and the antiprogrammed cell death 1 (PD1) inhibitor nivolumab. A repeat CT of the chest, abdomen, and pelvis prior to starting therapy showed worsening bilateral pleural effusions and peritoneal carcinomatosis compared to previous study.

**Figure 2:**
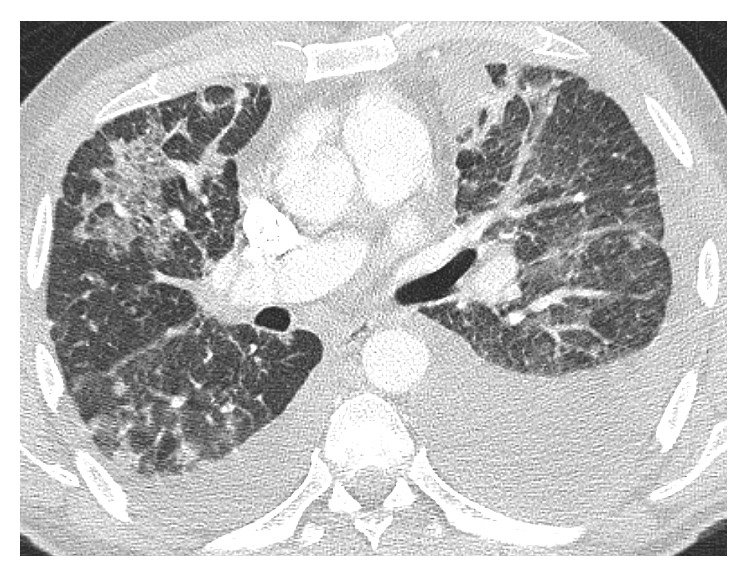
Chest CT showing scattered ground glass opacities and bilateral pleural effusions.

**Figure 3:**
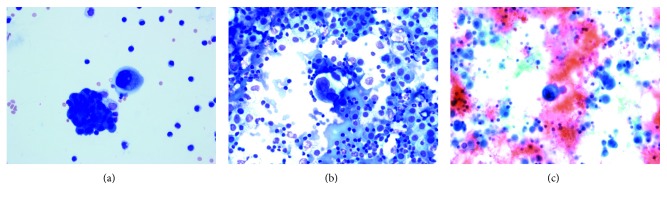
Pleural fluid involved by pleomorphic tumor cells. (a) Wright–Giemsa stain; (b) Romanowsky stain; (c) Papanicolaou stain, 400x.

While waiting for treatment initiation, he developed severe and rapid onset dyspnea which brought him in to the Emergency Department. An enhanced CT of the chest showed no evidence of acute pulmonary embolism, but innumerable soft tissue masses throughout both lungs with accompanying interlobular septal thickening were noted suggesting the possibility of lymphangitic spread. Bilateral pleural effusions with overlying atelectasis compared to the previous study. Axillary and mediastinal lymphadenopathy and hepatic metastases were unchanged from 10 days priorly (Figure 2). He was admitted to our hospital for further management and evaluation. He was empirically treated with antimicrobials to cover for a possible pneumonia. All infectious workup including blood cultures, *Streptococcus* antigen, and legionella testing were negative. Due to the size of the effusion, a left-sided thoracentesis was performed. The pleural fluid showed vacuolated tumor cells with hyperchromatic irregular nuclei with anisonucleosis and prominent nucleoli consistent with adenocarcinoma (Figure 3). On cell count, numerous lymphocytes were noted. An infectious workup of the pleural fluid, including gram stain and cultures, was negative. While hospitalized, it was decided that he should start on treatment promptly, as his clinical deterioration was likely due to disease progression.

He was discharged from the hospital to receive his first treatment with gemcitabine, nab-paclitaxel, and nivolumab as an outpatient. On that day, his transaminase and bilirubin levels were within normal range. Alkaline phosphatase was slightly elevated, and the serum albumin was slightly decreased. The white blood cell count was 20.1K/*µ*L (upper limit of normal 10.5K/*µ*L). CA19-9 was 4537 U/mL. Treatment was started as planned.

**Figure 4:**
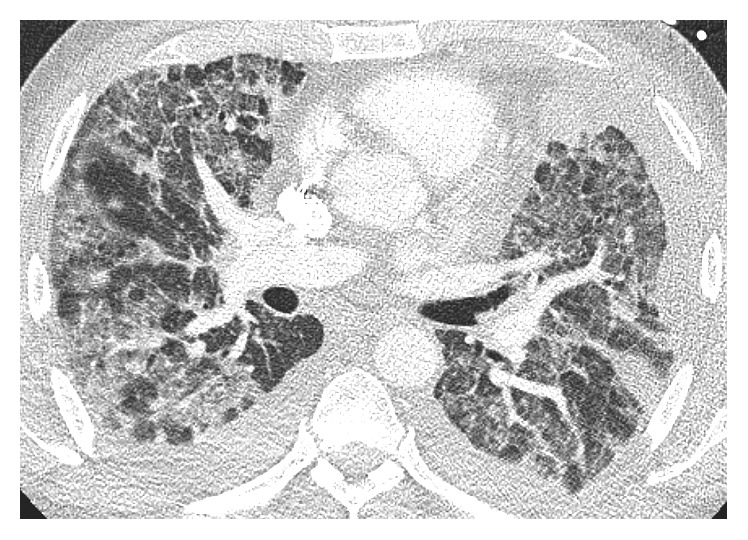
Chest CT showing diffuse ground glass opacities and new interlobular interstitial thickening.

Approximately eighteen hours after receiving his therapy, he woke up from sleep with increased dyspnea which prompted a visit to the ED. He was found to be hypoxemic with oxygen saturation around 80%. He was placed on BIPAP and transferred to the intensive care unit for further management. A CT angiogram of the chest revealed no pulmonary embolism at the lobar pulmonary artery branch level. There were progressive extensive ground glass opacities and interlobular septal thickening throughout the lungs compared to the previous study performed just four days priorly. There was a focal new airspace opacity in the posterior right upper lobe. The size of the pleural effusion was decreased on the left and stable on the right. There was no change in the extensive thoracic and abdominal metastatic lymphadenopathy (Figure 4). He was empirically treated with methylprednisolone and piperacillin/tazobactam for possible pneumonitis and infection. The steroids were continued throughout his hospital stay. On admission to the ICU, his labs were as follows: sodium 130 mEq/L (upper limit of normal (ULN) 135 mEq/L), potassium 5.6 mEq/L (ULN 5 mEq/L), creatinine 1.5 mg/dl (ULN 1.3 mg/dl), AST 3534 U/L (ULN 39 U/L), ALT 3206 U/L (ULN 52 U/L), total bilirubin within normal limits, INR 1.7, and WBC 21.9 (ULN 3.5K/*µ*L). A panel for respiratory viruses including influenza types A and B, RSV types A and B, parainfluenza types 1, 2, and 3, rhinovirus, metapneumovirus, and adenovirus types B, C, and E was negative. Blood cultures showed no growth. A viral hepatitis panel was negative. Four days later, his lab work was as follows: ALT 990 U/L, AST 173 U/L, albumin 2.5 g/dl, alkaline phosphatase 171, and total bilirubin 1.9 mg/dl. Six days from admission, his labs ALT 605 U/L, AST 83 U/L, albumin 2.4 g/dl, alkaline phosphatase 164 U/L, total bilirubin 2.5 mg/dl, INR 1.2, and creatinine 0.54 mg/dl.

After initial improvement, he had acute respiratory decompensation and opted for supportive care only. The family declined to have an autopsy performed.

## 3. Discussion

Pancreatic cancer is an aggressive neoplasm with a dismal prognosis as most patients present with metastatic disease. Despite recent therapeutic advances, one of its main characteristics is resistance to current therapies. The majority build upon chemotherapy backbones with other novel therapeutic strategies including immune and targeted therapies [6, 7].

Immunotherapy has shown promising activity for other neoplasms including lung, melanoma, bladder, and kidney [8–11]. Additionally, the toxicity profile of this class of drugs has consistently been shown to be acceptable. Grade 3-4 adverse events (AEs) occur in 7% to 12% with single-agent antiprogrammed cell (PD-1) monoclonal antibodies such as nivolumab. The most common AE is fatigue with an estimated rate of 16%–24% and grade 3-4 severity of 1-2%. Dermatological toxicity including rash, pruritus, and vitiligo has been reported in 34% of patients with a very low rate of grade 3-4 events with patients receiving nivolumab. Grade 3-4 colitis and hepatitis occur in approximately 1–3% of the cases. The incidence of grade 3-4 thyroid disorder is approximately 0.1%. Neurologic symptoms, ocular, pancreatic, and renal toxicity are rare events. Thus, toxicity rates have not been a major barrier for further development of this drug class [12–14].

Preliminary data with the use of the immune checkpoint inhibitor ipilimumab suggested that this approach could be further explored for the treatment of pancreatic adenocarcinoma [15]. Tumor shrinkage was documented in at least one patient with pancreatic cancer treated with the anti PDL1 antibody MEDI4376 after 6 weeks of treatment [16]. Clinical trials testing the combination of checkpoint inhibitors and chemotherapy are underway [7].

This patient was in excellent health and performance prior to his diagnosis of pancreatic cancer. Given previous results with standard therapy, enrollment in a clinical trial using a combination strategy was considered a reasonable option. Unfortunately, he developed acute severe respiratory failure after one single treatment despite meeting strict criteria for protocol initiation.

While an autopsy was not performed in this case, treatment-related lung injury was likely the cause of his hypoxemic respiratory failure. Viral or bacterial infection seems unlikely given results of cultures and previous evaluation. Decompensated heart failure was not diagnosed. The numerous lymphocytes in his pleural fluid after treatment with nivolumab may indicate an inflammatory response accounting for the acute presentation. Furthermore, his transaminases elevation is another substrate to support this diagnostic possibility.

One of the confounding factors here is that drug-induced lung injury has been well described with the use gemcitabine alone or in combination with taxanes. In a large database, the median time of diagnosis of severe lung toxicity was 48 days. However, symptoms were also seen within 1 day of administration of the drug [17]. Albeit uncommon, drug-related pneumonitis has also been reported with the use of single-agent nab-paclitaxel [18].

Pneumonitis has also been described with the use of checkpoint inhibitors including nivolumab with an estimated rate of approximately 2.5% for all grade toxicity and 0.8% for grade 3-4 toxicity. Its incidence appears to be higher with the use of combination treatment. The median time of onset is 2.5 months. It may present with different radiologic patterns. Initial treatment with methylprednisolone is usually successful [12, 19, 20]. Unfortunately, this patient received a combination of three drugs concurrently making it impossible to determine if a single, a doublet, or triplet combination were the culprit. The radiologic findings are nondiagnostic in this setting. Furthermore, the treatment of severe drug-induced pneumonitis usually consists of steroids with improvement. The lack of a specific radiologic pattern is also a characteristic of either immune- or chemotherapy-related pneumonitis. However, the fact that the transaminases promptly improved after institution of steroids also favors toxicity related to the immune checkpoint inhibitors. Severe transaminase elevations are scarce with the use of gemcitabine [21]. It is also possible that bronchoalveolar lavage and or transbronchial biopsy could have had elucidated the cause of respiratory failure. The fact that the previous cultures and infectious workup were previously negative made a weaker case for further interventions/procedures making the diagnosis elusive.

In summary, this case illustrates that pneumonitis with an immune checkpoint inhibitor-based combination may happen very rapidly despite careful selection of patients. Clinicians and patients should have a low threshold in considering pneumonitis with new respiratory symptoms.
